# The relationship between family social capital, social media use and life satisfaction in adolescents

**DOI:** 10.15171/hpp.2019.42

**Published:** 2019-10-24

**Authors:** Narjes Geraee, Ahmad Ali Eslami, Raheleh Soltani

**Affiliations:** ^1^Student Research Committee, School of Health, Isfahan University of Medical Sciences, Isfahan, Iran; ^2^Department of Health Education and Promotion, Isfahan University of Medical Sciences, Isfahan, Iran; ^3^Department of Health Education and Promotion, Arak University of Medical Sciences, Arak, Iran

**Keywords:** Adolescent, Satisfaction, Social capital, Social media, Structural equation modeling

## Abstract

**Background:** Nowadays, two social phenomena are identified as factors that significantly influence life satisfaction among adolescents: family social capital and social media use. This study aimed to investigate the direct and indirect relationships between family social capital and life satisfaction, and the possible mediating role of social media use between the variables among Iranian adolescents.

**Methods:** In 2018, this cross-sectional study was carried out on 835 adolescents aged from 12to 19, in six high schools of Isfahan, Iran. Data were collected using a validated four-section questionnaire including demographic characteristics (3 items), life satisfaction (5 items), family social capital (31 items) and social media use (4 items) scales. IBM SPSS version 21 statistical software and AMOS version 24 were used to analyze the data. Structural equation modeling was used to assess the fit of model. The significance level of 0.05 was considered for all data analyses.

**Results:** The findings indicated that family social capital and social media use explained 50%of the variance in life satisfaction. Social media use was found with a partial mediating role in the association between family social capital and life satisfaction. Family social capital was the strongest predictor of life satisfaction (β =0.681, P<0.001). The relationship between social media use and life satisfaction was also statistically significant (β =- 0.12, P<0.001).

**Conclusion:** Social media use and family social capital should be considered while investigating the determinants of life satisfaction among adolescents.

## Introduction

Life satisfaction is a cognitive component of subjective well-being,^1^ which reflects global and subjective assessments of an individual regarding his or her quality of life.^2^ Recently, life satisfaction has gained much attention as an indicator for optimal performance among adolescents.^3-6^ It may be considered as the psychological strength that helps adolescents to deal with different risks and challenges.^7^

Life satisfaction in adolescents is correlated with many family characteristics, such as family environment,^8^ family functioning,^9^ family composition,^10^ family cohesion,^11^ and family interactions.^12^ All of these characteristics may be encompassed as family social capital.^13^ On the other hand, social media have also gained increasing importance in the daily life of adolescents.^14^ It is, therefore, essential to study their possible effects on different aspects of well-being in adolescents.

Many studies have addressed the relationship between family social capital and life satisfaction.^15-18^ It is certain that family structure, functioning, and environment influence social media use among family members, especially adolescents.^19-21^ However, there is a scarcity in the studies that considered the mediating role of social media use while investigating the associations between family social capital and life satisfaction.

Previous studies have also investigated the effects of social media use on life satisfaction; however, their findings are inconsistent.^22^ Some studies have indicated a positive relationship between social media use and mental well-being, stating that using social media provides benefits for users with low self-esteem.^23,24^ In contrast, some studies have concluded that social media use has negative effects on life satisfaction in adolescents.^25-27^

**Figure 1:**
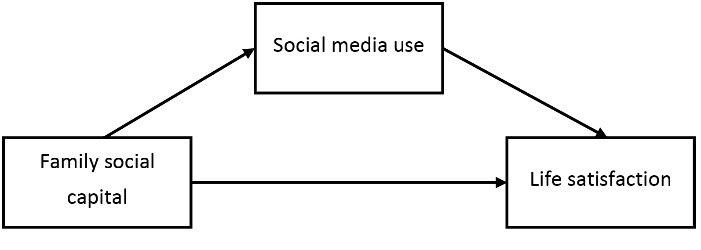
Hypothesized model of association between family social capital, social media use, and life satisfaction in adolescents.

Accordingly, the aim of this study was to investigate the relationships between family social capital, social media use and life satisfaction, and also to test the assumption that if the association between family social capital and life satisfaction may be mediated by social media use among adolescents (Figure 1).

## Materials and Methods

### Participants and procedure

In this cross-sectional study, data were collected from 835 adolescents. The mean age of participants was 15.15 (standard deviation = 1.73). Males constituted 51.3% of the participants. About one third of the participants belonged to under-supplied stratum (38.6%) (Table 1). Sample size was considered in accordance with the existing instructions regarding the sample size required for SEM studies.^28^ In order to have a representative sample of all socio-economic classes in Isfahan, stratified cluster sampling was used. Isfahan was divided into 3 strata based on a socioeconomic classification (well-supplied, moderately supplied, and under-supplied) conducted in a previous study in Isfahan.^29^ Expert opinions were also obtained to conduct the classification. Then, six high schools were randomly selected from each stratum (including both genders). Sample size was allocated to the selected school per the total number of students in each school. Finally, participants were selected via simple random sampling. Participants completed a pencil-and-paper questionnaire in schools and were informed about the voluntary nature of their participation.

### Scales

*Life satisfaction scale*

Life satisfaction was assessed using the satisfaction with life scale. It includes 5 items such as, “In most ways my life is close to my ideal” and “The conditions of my life are excellent”, with a seven-point Likert scale ranging from 1 (strongly disagree) to 7 (strongly agree). The possible score ranged from 5–35 and the higher score indicated the higher life satisfaction.^30^ It is a standard scale with proven reliability and validity and has been used in assessing overall life satisfaction in many different studies.^31-34^ Internal consistency estimate for the scale was 0.87 in the present study.

*Family social capital scale*

Family social capital scale was developed and validated in the present study. First, extensive literature review and expert interviews were conducted to explore the concept and identify questionnaire items. At the next step, a panel of experts was established and their views were used for qualitative and quantitative evaluation of the items by the means of two separate report forms for content validity index (CVI) and content validity ratio (CVR). For CVI index, the value 0.79 was considered to be acceptable.^35^ For CVR index, the value 0.52 was determined to be acceptable.^36^ In order to determine face validity and understandability of the items, the scale was submitted to 30 members of the target group. They were asked to state their opinion about the understandability of each item using a Likert scale consisting of the “completely understandable”, “understandable”, “relatively understandable” and “non-understandable” options. In addition, they were also asked to state their general opinions regarding the questionnaire and each item. After collecting their views, the impact score or the understandability of each item was determined. At this stage, no item was eliminated, and only items with spelling mistakes were corrected.

The following subscales were obtained via exploratory factor analysis: family cohesion (16 items), family interactions (9 items), lack of family conflicts (3 items), and family control (3 items). Response format for all items was based on a 5-point Likert-type scale ranging from 1 (strongly disagree) to 5 (strongly agree). The possible score range was 31–155 and the higher score indicated the higher level of family social capital. Construct validity was measured via confirmatory factor analysis (CFA). The fit indices were satisfactory (chi-square mean/degree of freedom (CMIN/df) =3.414, root mean square error of approximation (RMSEA) = 0.042, goodness of fit index = 0.939, adjusted goodness of fit index = 0.924, and all comparative indicators were above 0.9). The results of intergroup analysis and correlation analysis supported the validity of the developed scale. The reliability of the scale was confirmed based on internal consistency (Cronbach’s alpha for all subscales = 0.69–0.94).

*Social media use scale*

In the literature, there are various scales for measuring social media use with a rising trend in the field; however, there are growing concerns about their validity.^37^ As the use of social media varies according to the cultural context of each society,^38^ it seems essential to develop a native scale. To do so, extensive literature review and interviews with experts and adolescents were performed. Seven items were identified showing the most routine activities of adolescents while using social media platforms. After assessing content validity of the items applying CVI and CVR forms, three items were removed. The final scale consisted of the following four items: sending or receiving images, texts, videos, or music; checking the profile pictures, pages, posts and stories of others; Sharing daily routines with others; reading, commenting and liking others’ posts. The participants were asked to score their involvement level in these activities using a five-point Likert scale ranging from 1 (very low) to 5 (very high). The possible score range was 4–20, within which the higher score indicated the higher level of social media use. Face validity of the scale was confirmed by 30 adolescents. Exploratory and confirmatory factor analyses indicated the factor structure of the scale as unidimensional. The reliability of scale was approved (Cronbach’s alpha = 0.83).

### Statistics

The normality of data distribution was assessed by checking the skewness and kurtosis of the variables. All data analyses were performed in the IBM SPSS v.21 for Windows (IBM Corp., Armonk, NY, USA). Structural equation modeling (SEM) was used to analyze the relationships between life satisfaction, family social capital, and social media use. Data analysis was performed using IBM AMOS v.24 (IBM Corp., Chicago, IL, USA). In the SEM, the measurement model is used to identify the relationships between latent and manifest variables, and construct validity is examined by investigating four conditions: significance of factor loadings, factor loadings above 0.5, an average variance extracted (AVE) value above 0.5, a composite reliability (CR) value above AVE, and an AVE value above maximum shared variance (MSV) and average shared variance (ASV). Since the family social capital scale used in the study comprised four subscales, second-order CFA was utilized. According to Brown and Cudeck, the fit indices in this step were as follow: a RMSEA below 0.08, a parsimonious normed fit index above 0.5, Tucker-Lewis index (TLI), comparative fit index (CFI), and normed fit index above 0.9,^39^ a chi-square to degrees of freedom ratio (χ^2^/*df*) below 5.^40^ Considering the necessity to assess discriminant validity in the studies with latent variables,^41^ the Fornell and Larcker criterion was used to verify discriminant validity.^42^

In the next step, study hypotheses (latent variable relationships) were investigated using a structural model. The total R^2^ was checked to find out the extent to which family social capital and social media use collectively explained life satisfaction.

Finally, the bootstrapping method was utilized to determine the statistical significance of the indirect effects (i.e. mediated effects). This method is based on resampling and can result in 95% bias-corrected confidence intervals. If confidence intervals do not have a zero value (*P*< 0.05), then mediated effects are statistically significant.^43^

## Results

Means and standard deviations and skewness for all variables as well as Pearson’s r correlations between the variables are presented in Table 2. There was a significant positive correlation between family social capital and life satisfaction (*P*< 0.001). Social media use was negatively correlated with life satisfaction and family social capital (*P*< 0.001).

In the first order measurement model, all factor loadings were statistically significant and above 0.5 and the fit indices were acceptable (CMIN/df = 3.386, RMSEA = 0.053, CFI = 0.903, TLI = 0.896). In the second order measurement model, almost all factor loadings were significant and above 0.5. Goodness of fit was also acceptable (CMIN/df = 3.428, RMSEA = 0.054, CFI = 0.865, TLI = 0.894).

In order to examine construct validity of the model, four conditions and the Fornell and Larcker criterion were investigated. As there is shown in Table 3, all factor loadings were significant and above 0.5; the AVE was above 0.5; the CR was above AVE; and the AVE was above MSV and ASV, which all indicated the model with proper construct validity. The Fornell and Larcker criterion also confirmed the construct validity of the scales (the second part of Table 3, from SMU to FSC).

**Figure 2:**
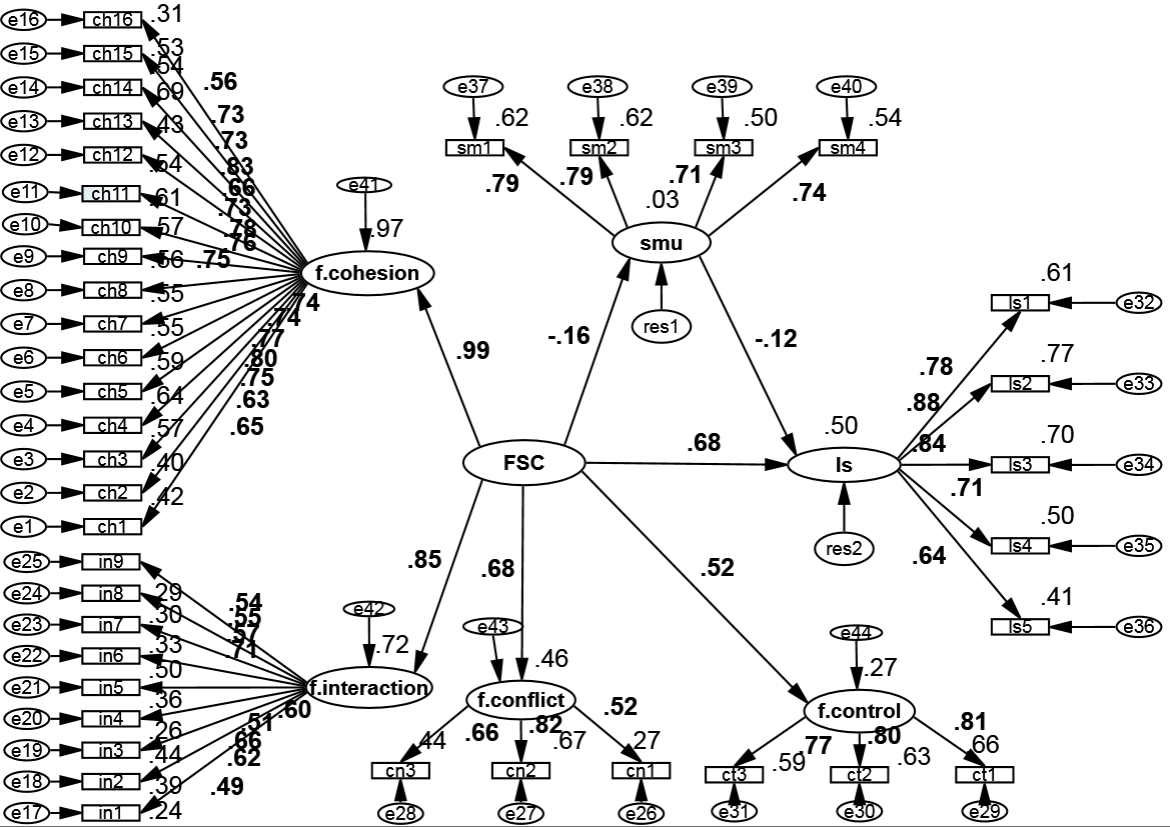
Direct and indirect path coefficients used for investigating the relationship between family social capital and life satisfaction, mediated by social media use. Note: f.cohesion= family cohesion, f.interaction= family interactions, f.conflict= lack of family conflict, f.control= family control, FSC= family social capital, SMU= social media use, LS= life satisfaction.

According to Figure 2 and Table 4, family social capital and social media use explained 50% of the total variance in life satisfaction (R^2^). Compared to social media use, family social capital was the stronger predictor for life satisfaction (β = 0.681, *P*< 0.001). The relationship between social media use and life satisfaction was also statistically significant (*P*< 0.001). The relationship, however, was weak and inverse as compared to family social capital (β = -0.117, *P*< 0.001). The relationship between family social capital and social media use was also statistically significant and inverse (β = -0.160, *P*< 0.001).

Table 5 shows the bootstrapped direct, indirect, and total effectsof family social capital on life satisfaction. Life satisfaction was directly affected by family social capital. Both direct and indirect effects were statistically significant (partially mediated). In other words, with the inclusion of social media use as a mediator, the path coefficient from family social capital to life satisfaction was still significant (c’= 0.681, *P*< 0.0001).

## Discussion

This study aimed to investigate the direct and indirect effect of family social capital on life satisfaction, and to assess the mediating role of social media use between the variables among adolescents in Isfahan, Iran. The results indicated social media use to be a statistically significant variable in the associations between family social capital and life satisfaction, that is, lower perceived family social capital was correlated with higher social media use, and higher social media use was in turn correlated with lower life satisfaction in adolescents. Family social capital and social media use explained 50% of the variance in life satisfaction in adolescents. Both family social capital and social media use were in statistically significant associations with life satisfaction among adolescents. However, the association of family social capital with life satisfaction was stronger than those between social media use and life satisfaction.

In line with previous studies, we also found that family social capital had a statistically significant positive association with life satisfaction in adolescents. Similar with our findings, Dubrov found that family social capital was a predictor of well-being among adolescents.^17^ Eriksson et al also reported that higher levels of family social capital were related to higher levels of well-being.^18^

Although adolescents spend a considerable amount of time with peers of the same age, family characteristics and attributes still have a stronger effect on their life satisfaction, which shows the necessity to determine the family characteristics that may be associated to their life satisfaction.^7^ The findings of this study indicated that family cohesion and family interactions had the strongest relationship with life satisfaction in adolescents. In line with our findings, previous studies also reported that life satisfaction and subjective well-being in adolescents are correlated with factors such as family cohesion,^11^ intra-family relationships,^44,45^ and parental support.^8^ Such studies clearly demonstrate that the quantity and quality of relationships within the family may have a detrimental role in satisfaction with family life among adolescents.

Contrary to some studies reporting the positive effects of social media use on life satisfaction and subjective well-being in adolescents,^23,24,46^ the findings of current study indicated a negative relationship between social media use and life satisfaction among adolescents. This finding, however, is in line with the findings of prior studies that indicated the negative effect of social media use on life satisfaction in adolescents.^47-49^ Some argue that such contradictions between the findings of different studies may be due to the ignorance of the usage type, stating that the users should be divided into two active and passive groups.^50^ While it is a noteworthy point, such conclusions are mostly related to adults who benefit from the ability of critical thinking,^51^ and may not be the case for adolescents who are mainly involved in emotional actions that are not fully based on thoughtful judgments.^52^

The results of our study also indicated a negative relationship between family social capital and social media use in adolescents (i.e. the lower the reported family social capital of adolescents, the higher the social media use). These results are in line with the findings of Mesch who reported that internet usage had a negative relationship with family closeness and a positive relationship with family conflicts.^53^ Lee also conducted a study on 1312 adolescents and found a negative correlation between the amount of adolescents’ online time and their interactions with their parents.^54^

### Limitations

Development of a comprehensive model for life satisfaction determinants in different age groups is quite difficult. It is clear that in the presumed model we presented in this study, we have not investigated many possible variables, including gender, age, socioeconomic status, mental and physical health and etc. Such variables could play different roles in the model, including confounding, mediating, or moderating, which need further investigations in future studies.

Another limitation of this study was its cross-sectional design, which did not allow us to report the causal interpretations of the findings. There may be reverse causality between some of the variables. For example, social media use can be both the cause and the effect of life satisfaction. Longitudinal studies are recommended to provide more reliable causative interpretations.

## Conclusion

In conclusion, social media use may have a partial mediator role in the association between family social capital and life satisfaction among Iranian adolescents. In future studies, social media use and family social capital should be considered while studying the determinants of life satisfaction among adolescents.

## Ethical approval

The study was approved by the Ethics Committee and Research Deputy of Isfahan University of Medical Sciences [code of ethics No IR.MUI.REC.1395.3.668]. The purpose of this study was explained to the participants, and a written consent was also obtained from participating students volunteered to enter this study.

## Competing interests

The authors declare that they have no competing interests.

## Funding

This work was supported by vice-chancellery of Research and Technology at Isfahan University of Medical Sciences, Isfahan, Iran, [grant No. 395668].

## Authors’ contributions

NG and AAE contributed to the design of the work, performed data collection and analysis, drafted the manuscript, performed significant revisions, and approved the final version of the manuscript. RS performed significant revisions, and approved the final version of the manuscript.

## Acknowledgments

The authors appreciate the vice-chancellery of Research and Technology at Isfahan University of Medical Sciences, Isfahan, Iran for their financial support. Special thanks to sincere contribution of adolescents who participated in this study.

**Table 1:** Demographic attributes of the participants

**Variables**	**Female**	**Male**	**Total**
Age, mean (SD)	15.16 (1.74)	15.14 (1.72)	15.15 (1.73)
Socioeconomic status, No. (%)			
Under-supplied	145 (44.8)	178 (55.1)	323 (38.6)
Moderately supplied	137 (50)	137 (50)	274 (32.8)
Well-supplied	109 (45.7)	129 (54.2)	238 (28.5)

**Table 2:** Descriptive data and variable relationships

**Variable**s	**LS**	**FSC**	**SMU**
LS	1		
FSC	0.615^a^	1	
SMU	-0.192^a^	-0.153^a^	1
M (SD)	24.51 (7.05)	119.88 (21.10)	12.88 (4.55)
Skewness	-0.633	-0.954	-0.164

Abbreviations: LS= life satisfaction, FSC= family social capital, SMU= social media use. ^a^ *P* < 0.001.

**Table 3:** Results of the construct validity examination

	**CR**	**AVE**	**MSV**	**ASV**	**SMU**	**LS**	**FSC**
SMU	0.842	0.571	0.051	0.038	0.756		
LS	0.880	0.598	0.489	0.270	-0.226	0.773	
FSC	0.853	0.605	0.489	0.257	-0.160	0.699	0.778

Abbreviations: CR= composite reliability, AVE= average variance extracted, MSV= maximum shared variance, ASV= average shared variance.

**Table 4:** Structure model for predicting social media use and life satisfaction

**Dependent**	**Predictor**	**B**	**SE**	**Beta**	***P***	**R** ^ 2 ^
SMU	FSC	-0.261	0.064	-0.160	<0.001	0.026
LS	SMU	-0.121	0.032	-0.117	<0.001	
LS	FSC	1.148	0.078	0.681	<0.001	0.502

Abbreviations: LS= life satisfaction, FSC= family social capital, SMU= social media use.

**Table 5:** Direct, indirect, and total effects of family social capital on life satisfaction with Bootstrapped confidence intervals

**Effect of X on M (a)**	**Effect of M on Y (b)**	**Indirect effect (ab)**	**Direct effect (c')**	**Total effect (c)**	**CI** ^a^	
					**Lower**	**Upper**
0.160^b^	-0.117^b^	0.019^b^	0.681^b^	0.699^b^	0.01	0.05

Abbreviations:X=exogenous variable; M= mediator; Y= endogenous variable; CI = confidence Interval. ^a^ Bias-corrected bootstrapping confidence intervals. ^b^ *P* < 0.0001.
